# PSMA-PET/CT-based salvage elective nodal radiotherapy for lymph node recurrence following radical prostatectomy

**DOI:** 10.1007/s00345-025-05950-5

**Published:** 2025-09-24

**Authors:** Samuel M. Vorbach, Hannah Rittmayer, Thomas Seppi, Bernhard Nilica, Mona Kafka, Ute Ganswindt

**Affiliations:** 1 Department of Radiation Oncology, Medical University of Innsbruck, Innsbruck, Austria; 2 Department of Nuclear Medicine, Medical University of Innsbruck, Innsbruck, Austria; 3 Department of Urology, Medical University of Innsbruck, Innsbruck, Austria

**Keywords:** PSMA-PET/CT, Prostate cancer, Nodal recurrence, Salvage elective nodal radiotherapy

## Abstract

**Purpose:**

For patients with oligometastatic nodal recurrence after radical prostatectomy (RP), salvage radiotherapy is a valuable curative second-line treatment option. However, few clinical data on the impact of PSMA-PET/CT-based salvage elective nodal radiotherapy (sENRT) is available. In order to contribute further clinical data on the outcome of patients treated with sENRT following RP, we analysed the rates of biochemical recurrence-free survival (BRFS) and distant metastasis-free survival (DMFS) as well as potential predictive markers for optimised patient selection.

**Methods:**

A retrospective analysis of 76 patients treated with sENRT for nodal recurrence after RP was performed. Primary endpoints were BRFS and DMFS. Cox proportional hazards model was used to analyse potential predictive factors.

**Results:**

Median follow-up was 32.6 months. PSMA-PET/CT revealed 1, 2, or ≥ 3 positive lymph nodes in 63.2%, 30.2% and 6.6% of patients, respectively. 96% of the patients had pelvic-only lymph nodes involvement. One-, two- and three-year BRFS were 98.6%, 84.2%, and 71.9%, respectively. Notably, nodal status at the time of RP and biochemical-recurrence prior to sENRT, were associated with reduced BRFS. One-, two-, and three-year DMFS were 98.7%, 94.1%, and 94.1%, respectively, with paraaortic lymph nodes being the only factor indicating reduced DMFS. Concomitant androgen-deprivation therapy was applied in 85.5% of the patients.

**Conclusion:**

We present one of the largest studies on PSMA-PET-based sENRT for nodal recurrence after RP with promising results, highlighting the important role of sENRT. Regarding patient selection, initial lymph node status and prior radiation of the prostate were predictive of reduced BRFS, while involvement of paraaortic lymph nodes was identified as marker for reduced DMFS.

## Introduction

 Prostate cancer (PCa) is the most common malignancy among men in Europe and the third leading cause of cancer-related deaths [1, 2]. While patients with localised PCa have high cure rates, this is no longer possible at the metastatic stage. Radical prostatectomy (RP) and external beam radiotherapy, with or without androgen deprivation therapy (ADT) depending on the risk group, are equal standard treatment options [3] in patients with local restricted PCa. However, biochemical recurrence (BCR) occurs in about one third of patients within 10 years after RP [4].

BCR after RP is defined as prostate-specific antigen (PSA) of >0.2 ng/ml three months after RP, while a PSA of ≥ 0.1 ng/ml immediately after RP is referred to as PSA persistence [3]. In this population, salvage radiotherapy (sRT) to the prostatic bed and lymphatics, if a lesion is detected, is a valuable second-line curative treatment or at least delays the time to metastatic progression. As CT scan or choline/fluciclovine PET/CT is mostly nonspecific when performed at low PSA levels, sRT was usually performed using a standardised field. With the introduction of prostate-specific membrane antigen positron emission tomography/computed tomography (PSMA-PET/CT), a novel tool is available for early detection of local recurrence and metastatic spreading with high sensitivity even at low PSA levels (detection rate of 38% at < 0.5ng/ml, 54% at < 1ng/ml) [5]. Thus, at present, treatment adjustments based on PSMA-PET/CT findings still need to be elaborated and standardised. Nevertheless, PSMA-PET/CT is already the recommended imaging modality in patients with BCR and PSA persistence and is increasingly used in primary staging, leading to a paradigm shift in PCa imaging [3]. For patients with formerly non-detectable nodal recurrence this novel imaging modality enables target-specific therapeutic approaches, either metastasis-directed therapy (MDT), including salvage lymph node dissection as well as stereotactic body radiotherapy (SBRT), or elective nodal therapy (ENRT) with a boost to the detected lesions.

However, there are still no clear recommendations regarding patient selection and treatment decisions, as randomised studies in this area are still very sparse. The recently published PEACE V-STORM trial is the first ever randomized study comparing MDT and ENRT in patients with oligometastatic nodal recurrences after curative treatment of localised PCa [6]. The study reports significantly higher biochemical recurrence-free survival (BRFS) and metastasis-free survival in the ENRT-treated cohort, suggesting superiority over MDT.

In order to contribute much-needed further clinical data on the outcome of patients treated with sENRT following RP, we aimed to analyse the rates of BRFS and distant metastasis-free survival (DMFS) in a large patient cohort. In addition, we sought to identify potential predictive markers for optimised patient selection. We also aimed to evaluate the role of concomitant androgen deprivation therapy in the sENRT concept.

## Patients and methods

### Study population

We retrospectively analysed 76 patients who underwent PSMA-PET/CT-based sENRT for nodal recurrence between 2015 and 2023 at the Department of Radiation Oncology, Medical University of Innsbruck. All patients had histologically confirmed PCa and were referred for sENRT after RP due to PSMA-PET/CT positive lymph nodes. Patients with prior RT of the prostate bed were included in our analysis. The extent of lymph node involvement was limited to the lumboaortic region below the renal arteries, classified as M1a disease.

### PSMA-PET/CT ligand and imaging protocol

All patients underwent pre-treatment imaging with 68Ga-labelled PSMA PET/CT (68Ga-PSMA-11). A GE DMI or GE Discovery 690 PET/CT (GE HealthCare, Chicago, IL, USA) scanner was used for PSMA-PET/CT imaging. At the time of the PET scan, either a contrast-enhanced diagnostic CT (120 kV, 100–400 mAs, with dose modulation) or a low-dose CT (120 kV, 25 mAs) was performed for attenuation correction. PSMA-PET/CT scans were obtained approximately 60 min after intravenous injection of the 68Ga ligand complex. PET/CT scans were interpreted in consensus by one nuclear medicine physician and one radiologist, each with more than ten years of PET/CT experience. PET positivity of lymph nodes was defined according to the PROMISE criteria [7].

### Radiotherapy treatment and follow-up

Patients received 3D conformal radiotherapy until 2017, after which volumetric modulated arc therapy was used. Daily cone beam CT was used for image guidance. A normofractionated regimen with a simultaneous integrated boost (SIB) to PET-positive lesions was applied. Target delineation followed the Radiation Therapy Oncology Group atlas [8], with adjustments based on PSMA-PET/CT findings. ADT was recommended for all patients for a period of 24 to 36 months, with duration individualised according to comorbidities, side effects and patient preferences. Follow-up assessments were initially performed at three-month intervals after RT and then at six-to-twelve-month intervals, depending on PSA progression.

### Endpoint and statistical analysis

The primary endpoints were biochemical recurrence-free survival defined as PSA < post-RT nadir + 0.2 ng/ml and distant metastasis-free survival. PSMA-PET/CT for the detection of metastases was performed in case of biochemical recurrence, and in the case of a negative scan, further imaging was conducted at the discretion of the treating urologist if PSA values continued to rise. Survival data were calculated from last sENRT session to biochemical progression / diagnosis of distant metastases or last follow-up date. Statistical analysis was performed using SPSS Statistics (V26, IBM Cooperation, Armonk, NY, USA) and GraphPadPrism (V10, GraphPad Software Inc., San Diego, CA, USA). Descriptive analysis was used to summarise relevant primary patient and treatment characteristics. The Kaplan-Meier method was used to calculate BRFS and DMFS. The Cox proportional hazards model was used for the univariate and multivariate analysis of predictive factors. Multivariate analysis was performed using the rule of stepwise backward elimination of non-significant factors. The level of significance was defined as P values < 0.05.

## Results

### Patient population

Between May 2015 and October 2023, 76 patients underwent PSMA-PET/CT sENRT for nodal recurrence after RP. Indication for PSMA-PET CT was BCR in all patients and the median follow-up after sENRT was 32.6 months. Initial tumour stage was ≥ pT3 in 63.2% (47 patients) with the majority of patients (72.4%) having an International Society of Urological Pathology (ISUP) score of 3 or higher. 79% of the patients had an initial negative nodal stage (pN0), and 27.6% had positive surgical margins (R1). The median PSA at RP was 6.83 ng/mL (range: 2.00–73.6 ng/mL), with a median post-RP PSA level of 0.01 ng/mL (range: 0.00–0.03 ng/mL). A subpopulation of 31.6% (24 patients) had received prostate bed radiotherapy for BCR prior to sENRT.

The median time from RP to nodal recurrence was 44 months (range: 2–158 months). Median PSA at the time of PSMA-PET/CT was 0.46 ng/mL (range: 0.02–10.09 ng/mL). PSMA-PET/CT revealed one, two, or three or more positive lymph nodes in 63.2% (48 patients), 30.2% (22 patients) and 6.6% (5 patients) of patients, respectively. The majority of patients in our cohort had pelvic-only lymph node metastases (96.1%). Two patients had paraaortic lymph node metastases only (2.6%), and one patient (1.3%) had both paraaortic and pelvic lymph node metastases. In addition, 14 patients (18.4%) had local recurrence detected by PSMA-PET/CT (Table 1).

**Table 1:** Patient characteristics

Characteristic	Value
Total no. of patients	76
Age at the time of sENRT, years Median (range)	70 (57–82)
*Initial tumour stage*	
pT2a	5 (6.6%)
pT2b	4 (5.2%)
pT2c	19 (25.0%)
pT3a	31 (40.8%)
pT3b	16 (21.1%)
pT4	1 (1.3%)
*Initial nodal stage*	
pN0	60 (79.0%)
pN1	8 (10.5%)
pNx	8 (10.5%)
*ISUP score*	
1	2 (2.6%)
2	19 (25.0%)
3	27 (35.5%)
4	12 (15.8%)
5	16 (21.1%)
PSA at RP, ng/mL Median (range)	6.83 (2.00–73.57)
*Surgical margins*	
R0	55 (72.4%)
R1	21 (27.6%)
Age at the time of RP, years Median (range)	64 (52–76)
PSA post RP, ng/mL Median (range)	0.01 (0.00–0.03)
Patients with RT to prostate bed before sENRT for prior BCR	24 (31.6%)
Time between RP and RT to prostate bed, months Median (range)	10 (1–72)
Time between RT to prostate bed and sENRT, months Median (range)	45 (11–154)
Time between RP and nodal recurrence, months Median (range)	44 (2–158)
PSA at PSMA-PET/CT, ng/ml Median (range)	0.46 (0.02–10.09)
*Number of PSMA-PET/CT-positive lymph nodes*	
1	48 (63.2%)
2	22 (30.2%)
≥ 3	5 (6.6%)
*Localisation of PSMA-PET/CT-positive lymph nodes per patient*	
Pelvic-only	73 (96.1%)
Paraaortic	2 (2.6%)
Paraaortic + pelvic	1 (1.3%)
Patients with local recurrence on PSMA-PET/CT	14 (18.4%)

*sENRT* salvage elective nodal radiotherapy, *ISUP* International Society of Urological Pathology, *PSA* prostate-specific antigen, *RP* radical prostatectomy, *BCR* biochemical recurrence

Most patients (85.5%) received concomitant ADT during sENRT, while 11 patients (14.5%) refused ADT despite recommendation (Table 2). The majority (67.7%) received ADT for ≥ 24 months and only 3 patients (4.6%) had ADT for less the 6 months. 53 patients (69.7%) were not on ADT at the last follow-up and the median time without ADT for these patients was 25 months (range 3–101).

Median RT doses converted to EQD2 equivalent using an α/β ratio of 1.5 Gy were 67.9 Gy to the prostate bed (range 62.8–70.1 Gy), 70.0 Gy to local recurrence, if present, (range 66–72 Gy). Elective lymph node regions received a median dose of 47.5 Gy (range 42.4–47.5 Gy), and PSMA-PET/CT positive lymph nodes received a simultaneous integrated boost of median 62.3 Gy (range: 55.6–67.5 Gy).

**Table 2:** Treatment characteristics

Characteristic	Value
*ADT*	
No ADT	11 (14.5%)
Concomitant ADT	65 (85.5%)
*ADT duration*	
< 6 months	3 (4.6%)
6–12 months	3 (4.6%)
13–23 months	15 (23.1%)
≥ 24 months	44 (67.7%)
Follow-up time without ADT, months Median (range)	25 (3–101)
Dose to prostate bed EQD2_1.5_, Gy Median (range)	67.9 (62.8–70.1)
Dose to local recurrence EQD2_1.5_, Gy Median (range)	70.0 (66.0–72.0)
Dose to elective lymph node regions EQD2_1.5_, Gy Median (range)	47.5 (42.4–47.5)
Dose to PSMA-PET/CT-positive lymph nodes EQD2_1.5_, Gy Median (range)	62.3 (55.6–67.5)

**Fig. 1:**
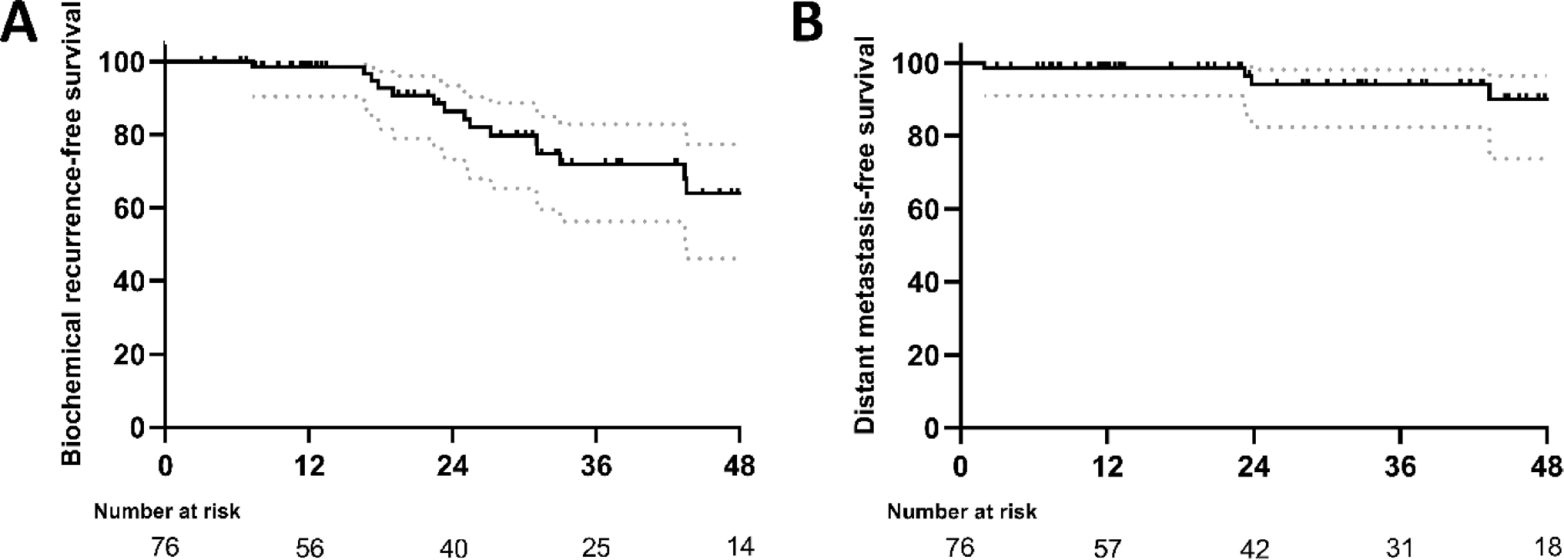
Kaplan-Meier curve showing (a) biochemical recurrence-free survival and (b) distant metastasis-free survival in months (dotted lines: 95% confidence interval)

### Treatment outcome

At the end of follow-up, the median BRFS was not reached, with the majority of patients (77.6%) free of BCR. One-, two- and three-year BRFS were 98.6% (95% CI: 90.4 to 99.8%), 84.2% (95% CI: 70.6 to 91.8%) and 71.9% (95% CI: 56.2 to 82.9%), respectively. In patients without ADT at last follow-up, the one-, two- and three-year BRFS were 100.0% (95% CI: 100 to 100.0%), 87.0% (95%CI: 73.3 to 93.9%) and 72.5% (95%CI: 56.5 to 83.4%), respectively. 

Prognostic factors associated with decreased BRFS in the univariate analysis were PSA nadir (≥ 0.5 ng/ml vs. < 0.5 ng/ml after sENRT, HR = 8.06, 95% CI: 2.78–23.35, *p* < 0.001, Table 3), initial nodal stage of N1 or Nx (vs. N0, HR = 3.16, 95% CI: 1.24–8.07, *p* = 0.016), and BCR prior to sENRT (vs. none, HR = 5,17, 95% CI: 1.92–13.88, *p* < 0.001). Initial nodal stage (N1 + Nx vs. N0, HR = 6.92, 95% CI: 2.25–21.23, *p* < 0.001) and BCR prior to sENRT (HR = 9.83, 95% CI: 3.05–31.74, *p* < 0.001) were confirmed to be significantly associated with BRFS in multivariate analysis. The PSA nadir after sENRT is correlated with reduced BRFS in the multivariate analysis (≥ 0.5 ng/ml vs. < 0.5 ng/ml, HR = 3.47, 95% CI: 0.97–12.41, *p* = 0.055), but without statistical significance

The median DMFS was not reached as only 5 patients (6.5%) developed bone or lymph node metastases during follow-up. One-, two- and three-year DMFS were 98.7% (95% CI: 91.0 to 99.8%), 94.1% (95% CI: 82.4 to 98.0%), and 94.1% (95% CI: 82.4 to 98.0%), respectively. In patients without ADT at last follow-up, the one-, two- and three-year DMFS were 98.1% (95% CI: 87.3 to 99.7%), 95.8% (84.0 to 99.4%), and 95.8% (84.0 to 99.4%), respectively.

Paraaortic lymph node metastasis was the only prognostic factor significantly associated with reduced DMFS (vs. pelvic only, HR = 17.32, 95% CI: 1.54–194.63, *p* = 0.021, Table 4)

**Table 3:** Univariate and multivariate analysis of risk factors related to BRFS

Factor	Univariate hazard ratio for BRFS		Multivariate hazard ratio for BRFS	
	HR (95% CI)	*p* Value	HR (95% CI)	*p* Value
*Initial tumour stage*				
T2	1 (reference)			
≥ T3	0.55 (0.21–1.43)	0.218		
*Initial nodal stage*				
N0	1 (reference)		1 (reference)	
N1 + Nx	3.16 (1.24–8.07)	**0.016**	6.92 (2.25–21.23)	**< 0.001**
*ISUP score*				
≤ 3	1 (reference)			
≥ 4	0.65 (0.24–1.74	0.394		
*Biochemical recurrence prior to sENRT*				
No	1 (reference)		1 (reference)	
Yes	5.17 (1.92–13.88)	**< 0.001**	9.83 (3.05–31.74)	**< 0.001**
*Number of lymph node metastases*				
1	1 (reference)			
≥ 2	1.38 (0.54–3.54)	0.499		
*Lymph node localisation*				
Pelvic	1 (reference)			
Paraaortic (± pelvic)	2.83 (0.37–21.86)	0.319		
*Concomitant ADT*				
Absent	1(reference)			
Present	0.56 (0.16–1.99)	0.370		
*ADT duration*				
< 24 months	1 (reference)			
≥ 24 months	1.07 (0.42–2.71)	0.889		
*PSA before sENRT*				
< 0.5 ng/ml	1 (reference)			
≥ 0.5 ng/ml	2.14 (0.75–6.09)	0.154		
*Local recurrence*				
No local recurrence	1 (reference)			
Local recurrence	0.79 (0.18–3.46)	0.741		
*PSA nadir post sENRT*				
< 0.5 ng/ml	1 (reference)		1 (reference)	
≥ 0.5 ng/ml	8.06 (2.78–23.35)	**< 0.001**	3.47 (0.97–12.41)	0.055

*CI* confidence interval, *ISUP* International Society of Urological Pathology, *ADT* androgen deprivation therapy, *PSA* prostate-specific antigen, *sENRT* salvage elective nodal radiotherapy; p-values < 0.05 are shown in bold

**Table 4:** Univariate analysis of risk factors related to DMFS

Univariate hazard ratio for DMFS		
Factor	HR (95% CI)	*P* Value
*Initial tumour stage*		
T2	1 (reference)	
≥ T3	0.64 (0.11–3.88)	0.630
*Initial nodal stage*		
N0	1 (reference)	
N1 + Nx	0.77 (0.09–6.88)	0.811
*ISUP Score*		
≤ 3	1 (reference)	
≥ 4	0.34 (0.04–3.18)	0.343
*Biochemical recurrence prior to sENRT*		
No	1 (reference)	
Yes	2.83 (0.47–16.94)	0.255
*Number of lymph node metastases*		
1	1 (reference)	
≥ 2	0.39 (0.04–3.52)	0.402
*Lymph node localization*		
Pelvic	1 (reference)	
Paraaortic (± pelvic)	17.32 (1.54–194.63)	**0.021**
*Concomitant ADT*		
Absent	1(reference)	
Present	0.81 (0.00–70.50)	0.632
*ADT duration*		
< 24 months	1 (reference)	
≥ 24 months	1.71 (0.28–10.50)	0.557
*PSA before sENRT*		
< 0.5 ng/ml	1 (reference)	
≥ 0.5 ng/ml	0.91 (0.14–6.09)	0.923
*Local recurrence*		
No local recurrence	1 (reference)	
Local recurrence	1.92 (0.20–18.80)	0.576
*PSA nadir post sENRT*		
< 0.5 ng/ml	1 (reference)	
≥ 0.5 ng/ml	0.39 (0.01–76.72)	0.552

*CI* confidence interval, *ISUP* International Society of Urological Pathology, *ADT* androgen deprivation therapy, *PSA* prostate-specific antigen, *sENRT* salvage elective nodal radiotherapy; *p*-values < 0.05 are shown in bold

## Discussion

Our study includes one of the largest published collectives of patients treated with PSMA-PET/CT-based sENRT for nodal-only recurrence after RP. Although our patients are part of a high-risk cohort, with 63.2% initially staged as ≥ pT3 and 72.4% with an ISUP grade of ≥ 3 at the time of RP, we have achieved promising results, with a one-, two- and three-year BRFS rate of 98.6%, 84.2% and 71.9%, respectively. The median BRFS was not reached at the end of follow-up (32.6 months), as 77.6% of patients were without BCR.

Our analysis of DMFS also showed desirable results with a one-, two-, and three-year DMFS of 98.7%, 94.1%, and 94.1%, respectively. Again, the median DMFS was not reached with only five patients developing bone or lymph node metastases.

As the median PSA at the time of PSMA-PET/CT was 0.46ng/ml, our results further confirm the high sensitivity of PSMA-PET/CT at low PSA levels and support the clinical decision for early imaging to preserve the option of additional curative treatment at the earliest stage.

Regarding our search for prognostic markers, we found that initial pN1/Nx status (*p* = 0.016) and a prior prostate bed irradiation to treat earlier occurring BCR (*p* < 0.001) were factors correlated with worse BRFS. These parameters should be considered when selecting patients for sENRT and for treatment intensification with long-term ADT.

As expected, paraaortic localisation of affected lymph nodes (*p* = 0.021) was associated with reduced DMFS. This underscores the higher risk of more rapid metastatic progression in these patients, advocating close follow-up and rapid treatment intensification towards a dual systemic therapy if necessary. Remarkably, neither initial T stage, total number of positive lymph nodes, PSA prior to sENRT, nor the use or duration of ADT had a prognostic impact on BRFS or DMFS in our cohort.

In summary, our results surpass the already desirable findings of a previously published retrospective study by Rogowski et al. [9]. In fact, the authors report one-, two- and three-year BRFS rates of 80.7%, 71.6% and 65.8%, and one-, two- and three-year DMFS rates of 91.6%, 76.9% and 63.6% with a median follow-up of 37.6 months. In contrast to our study, they did not only include patients with nodal recurrence after RP (24%), but mainly patients with PSA persistence (76%) after RP, a subgroup with known worse prognosis compared to patients with BCR only [10, 11]. This fact is reflected by the significantly longer period between RP and sENRT in our cohort (44 months versus 22.5 months). In addition, PSMA-PET/CT in our cohort was performed at lower PSA-levels (0.46 ng/ml versus 1.7 ng/ml), enabling prompt sENRT.

A further reason for lower BRFS and DMFS in the study of Rogowski et al. might be the inclusion of more patients with paraaortic nodal recurrence (17 versus 3 patients), which is reflected also by our finding of paraaortic lymph node involvement being predictive for impaired DMFS.

The total number of patients receiving concomitant ADT is comparable in both studies. We did not detect any correlation between ADT administration and BRFS or DMFS, likely due to the prevailing ADT long-term administration in our patients (≥ 12 months: 90.8% versus 39% reported by Rogowski et al.). Nevertheless, the different outcomes might in addition be explained by the overall shorter administration of ADT in the cohort of Rogowski et al., as they accordingly reported that a longer duration of concomitant ADT was correlated with better BRFS.

Tamihardja et al. showed in their study with 95 patients treated with sENRT for nodal recurrence after initial RP or radiotherapy, that 75% of patients not receiving concomitant ADT experienced a biochemical progression within 5 years after sENRT compared to 35.3% with ADT administration [12]. Compared to our study, they observed generally higher progression rates with 35.8% of their patients experiencing biochemical progression and 27.4% developing distant metastases within 5 years. However, only 50% of their patients received outcome-favourable PSMA-PET/CT scans to detect nodal recurrence readily.

The low rate of PSMA-PET/CT imaging might also be the reason of a moderate BRFS rate of 53% at 3 years after sENRT reported by another smaller study [13]. In fact, for 88% of their patients, sENRT-planning was based on findings from choline-PET/CT, whereas only 5% underwent PSMA-PET/CT. Consequently, results from other studies [14] using imaging modalities other than PSMA-PET/CT are difficult to compare with our investigation.

A multicentre retrospective study by Trapp et al. compared whole pelvis versus hemi-pelvis PSMA-PET/CT-based sENRT, demonstrating comparable outcomes [15]. Alternatively, Werensteijn et al. report on a MDT approach using PSMA-PET/CT-directed SBRT without concomitant ADT for the treatment of lymph node oligometastases, achieving a median BRFS of 21 months [16]. As MDT continues to expand in the management of PCa, PSMA-PET/CT-guided salvage lymph node dissection is increasingly performed. However, current literature does not show an advantage over sENRT, since DMFS is reported to be similar or even impaired [17, 18]. These studies show quite clearly that there is more than one promising approach to the treatment of nodal recurrence in PCa and, where appropriate, a standardised approach to treatment still needs to be developed.

In response to the question of whether MDT or ENRT is the preferable treatment modality, the results of the first randomised phase-II trial were recently published [6]. The PEACE V-STORM trial randomised 196 patients with oligometastatic pelvic recurrences after RP, detected by choline or PSMA-PET/CT, into two groups. One group received MDT (including lymphadenectomy and SBRT), while the other received ENRT (with a boost to the detected lesions), both with six months of ADT. A significantly longer metastasis-free survival was observed in the ENRT group (4-year MFS: ENRT 76% versus MDT 63%), thereby comparing favourably with the recently published phase-II GETUG-P07–OLIGOPELVIS trial (using choline as PET tracer) [19], in which the 3-year BRFS for ENRT was 45% and thus substantially lower than in both, the ENRT and even the MDT group of the PEACE V-STORM trial.

To conclude, and in accordance with our results, PSMA-PET/CT-based sENRT proved to be effective, even if combined with short-term ADT, and might become the standard treatment approach upon further investigation in a phase-III trial [6, 20].

Finally, our study has some limitations: (I) the study is retrospective and includes a representative cohort of patients with nodal-only recurrence with or without prior RT for BCR; (II) neither recordings of treatment-related toxicity nor of quality-of-life assessment are available; and (III) even if the follow-up time is comparable to similar studies, a longer follow-up would be desirable to report on additional endpoints such as overall survival.

## Conclusion

 To our knowledge, this analysis presents one of the largest studies to date on the impact of PSMA-PET/CT-based sENRT in the PSMA-PET/CT era. Our results clearly demonstrate that PSMA-PET/CT should be performed at low PSA levels to enable the earliest possible detection of recurrence, thereby increasing the likelihood of successful curative second-line treatment. Our follow-up shows that early PSMA-PET/CT-based sENRT for nodal recurrence translates into a three-year BRFS of 71.9% and a three-year DMFS of 94.1%. We identified involved lymph nodes at the time of RP and prior RT to the prostate bed as correlated with reduced BRFS. In addition, paraaortic lymph node metastasis predicted reduced DMFS, suggesting that these patients may be at significantly higher risk for faster metastatic progression and may not be the ideal candidates for sENRT. Consequently, the possibility of intensifying treatment towards a dual systemic therapy should at least be further discussed.

## Data Availability

The data presented in this study are available upon reasonable request from the corresponding author.
